# Extensive ITR expansion of the 2022 Mpox virus genome through gene duplication and gene loss

**DOI:** 10.1007/s11262-023-02002-1

**Published:** 2023-05-31

**Authors:** Annika Brinkmann, Claudia Kohl, Katharina Pape, Daniel Bourquain, Andrea Thürmer, Janine Michel, Lars Schaade, Andreas Nitsche

**Affiliations:** 1 Centre for Biological Threats and Special Pathogens, WHO Collaborating Centre for Emerging Infections and Biological Threats, Highly Pathogenic Viruses, German Consultant Laboratory for Poxviruses, Robert Koch Institute, Seestraße 10, 13353 Berlin, Germany; 2 Genome Sequencing and Genomic Epidemiology, Methodology and Research Infrastructure, Robert Koch Institute, Seestraße 10, 13353 Berlin, Germany

**Keywords:** Mpox virus 2022, Illumina sequencing, Poxvirus ITR, Mpox virus genome, Gene duplication

## Abstract

Poxviruses are known to evolve slower than RNA viruses with only 1–2 mutations/genome/year. Rather than single mutations, rearrangements such as gene gain and loss, which have been discussed as a possible driver for host adaption, were described in poxviruses. In 2022 and 2023 the world is being challenged by the largest global outbreak so far of Mpox virus, and the virus seems to have established itself in the human community for an extended period of time. Here, we report five Mpox virus genomes from Germany with extensive gene duplication and loss, leading to the expansion of the ITR regions from 6400 to up to 24,600 bp. We describe duplications of up to 18,200 bp to the opposed genome end, and deletions at the site of insertion of up to 16,900 bp. Deletions and duplications of genes with functions of supposed immune modulation, virulence and host adaption as B19R, B21R, B22R and D10L are described. In summary, we highlight the need for monitoring rearrangements of the Mpox virus genome rather than for monitoring single mutations only.

**Supplementary Information:**

The online version contains supplementary material available at 10.1007/s11262-023-02002-1.

## Introduction

Mpox virus (MPXV) is a species within the genus Orthopoxvirus (OPXV). Beside MPXV the genus OPXV comprises other OPXV like camelpox virus (CMLV), cowpox virus (CPXV), vaccinia virus (VACV) and variola virus (VARV). Following the eradication of the latter, MPXV was described as the most important OPXV species for humans, and in the years following VARV eradication concern was raised that MPXV might fill the vacant epidemiological niche of VARV [1–3]. However, between the first identified case of human Mpox in 1970 in the Democratic Republic of the Congo (DRC) and the first outbreak in a previously not affected country with 81 identified cases (in 2003, USA), only few cases of human Mpox were reported outside the endemic region of DRC [4, 5]. In the following years, further outbreaks were reported in the Sudan (2005) and in several countries, e.g. Nigeria, Cameroon and Liberia, in Central and West Africa (2017–2018), some of which had not reported any cases of human Mpox for almost 40 years [6–8]. In 2018 and 2019, cases connected to travelers infected in Nigeria were reported outside of Africa for the second time in the UK, Israel and Singapore [9–11]. Not only had Mpox been “reported from more countries in the past decade than during the previous 40 years” [12], but also human cases had more than doubled in that period. However, up to this point, no more than seven serial human transmission events had been reported, and MPXV had been considered unlikely to maintain itself in human communities without repeated zoonotic spill-over events from its (yet unknown) natural host [13–18].

In May 2022, cases of Mpox were reported in the UK, followed by Spain, Portugal, Germany, the US and several countries all over the world [19–21]. On 20 January 2023, 84,916 cases were reported in 110 countries, 103 of which had never reported Mpox before [22]. Total deaths were 81, the most affected countries being the United States (21), Brazil (14), Peru (12), Nigeria (7), Mexico (4), Ghana (4), Spain (3) and Cameroon (3). Phylogenetically, the current outbreak strain belongs to clade II (former West African clade) generally considered to be less virulent than clade I (former Congo Basin clade) [23]. The current strain forms a divergent branch (clade IIb) with 46 nucleotide differences to Nigerian strains from 2019 and 16 nucleotide differences to a strain from a patient travelling from Nigeria to the USA in 2021 [24, 25]. The high mutation rate in the current outbreak strain, which was previously estimated as 1–2 nucleotides/genome/year for poxviruses and the distinct signature of nucleotide changes (GA > AA and TC > TT), may suggest human APOBEC cytosine deaminase enzyme activity and previous or ongoing adaption to the human host [24, 26]. However, awareness has been raised to not only survey single nucleotide polymorphisms, but rather monitor integrity and stability of the MPXV genomic termini [27]. The double-stranded DNA genome of MPXV contains identical but oppositely oriented sequences (inverted terminal repeats, ITR) of ~ 6400 bp in length [28]. While essential genes for replication are present in the central, highly conserved core of the genome, the diverse terminal regions of the genome contain genes assumed to be involved in immune evasion and host range [29, 30]. Gene gain and loss in the terminal regions are described to be drivers of poxvirus evolution and adaption to the host [31, 32]. Deletions in the genomes of clade I MPXV in DRC were correlated with human-to-human transmission [33], and gene copy number variation has been described as a factor for modulating viral fitness [34]. Furthermore, in VARV, gene loss has been associated with restricted host range, but not severity of disease [35].

As the consultant laboratory for poxviruses in Germany, one of the most affected countries in Europe, we have received a broad variety of MPXV-positive samples. We have closely monitored the outbreak on genome level. So far, 339 genomes were sequenced. Out of these 339, five genomes displayed a similar but highly diverse genomic setup. Here, we report five MPXV genomes from Germany belonging to the multi-country 2022 outbreak, with duplication of several consecutive genes of up to 18,200 bp from the left to the right ITR region as well as from the right to the left ITR region, resulting in gene deletions of up to 16,900 bp in the region of insertion and expansion of the ITR from 6400 to up to 24,600 bp. We discuss gene duplication and loss as possible mechanisms of adaption to the human host in the current Mpox outbreak and highlight the need for surveillance of the genome ends rather than or in addition to ongoing monitoring of non-synonymous mutations.

## Material and methods

We subjected 339 samples screened positive for MPXV by qPCR to Illumina sequencing. All samples were submitted for MPXV diagnostics from patients in the Berlin area. Only limited patient metadata were available. DNA was extracted using the QIAamp viral RNA mini kit (Qiagen). Whole genomes shotgun libraries were prepared using the Nextera XT Kit (Illumina). Samples were sequenced on the NextSeq 2000 with approximately 10–30 million reads per sample, using P2 chemistry with 2 × 150 bp. Human background reads were removed, mapping to H. sapiens GRCh38 with bowtie2 v2.3.0. Genomes were constructed with de novo assemblies using Spades v3.13.1 and mapping to a reference sequence (Monkeypox/PT0006/2022|sampling_date_20220515, accession number ON585033.1). Alignments were prepared with MAFFT V7.307. All genomes in this study were uploaded to NCBI GenBank (MPXV/Germany/2022/RKI01 – MPXV/Germany/2022/RKI339, described in this study with rearrangements: MPXV/Germany/2022/RKI335—MPXV/Germany/2022/RKI339), accession numbers OP696838, OP696839, OP696840, OP696841 and OP696842. All sequence data are deposited under the project number PRJEB56807 at the European Nucleotide Archive (Table 1).

**Table 1:** The five MPXV genomes with genetic rearrangements discussed in this study are derived from samples from five individual men from the Berlin area, aged 30–52 with unknown courses of disease and immune status. All samples were obtained by swabbing skin lesions

#	Sex	Age	Lesion	Date of sampling	Reported symptoms	Travel history	Accession	Sample ID
1	m	32	Penis, primary lesion	5th July 2022	Fever, back pain, lymphadenopathy	Italy	OP696838.1	MPXV/Germany/2022/RKI335
2	m	31	Penis, wound /ulcus	17th June 2022	Unknown	n/d	OP696839.1	MPXV/Germany/2022/RKI336
3	m	46	Lesion, region not reported	13th June 2022	Lymphadenopathy	None	OP696840.1	MPXV/Germany/2022/RKI337
4	m	52	Lesion, region not reported	21st July 2022	Unknown	None	OP696841.1	MPXV/Germany/2022/RKI338
5	m	37	Lesion, region not reported	5th September 2022	Unknown	Unknown	OP696842.1	MPXV/Germany/2022/RKI339

The sequential RKI identifier numbers are due to simultaneous upload, while the samples have been sequenced on different timepoints and NGS plates.

Sample MPXV/Germany/2022/RKI335 was obtained from a male patient aged 32 from Berlin, with fever, back pain and lymphadenopathy, swabbed from a primary lesion on the penis on 5 July 2022. The patient reported a supposed travel-related infection after visiting Italy. Sample MPXV/Germany/2022/RKI336 was obtained from a male patient aged 31 from Berlin with unknown symptoms and without travel history, swabbed from a wound/ulcus on the penis on 17 June 2022. Sample MPXV/Germany/2022/RKI337 was obtained from a male patient aged 46 with reported lymphadenopathy and no travel history, swabbed from a lesion, region not reported, on 13 June 2022. Sample MPXV/Germany/2022/RKI338 was obtained from a male patient aged 52 with unknown symptoms and no travel history, swabbed from a lesion, region not reported, on 21 July 2022. Sample MPXV/Germany/2022/RKI339 was obtained from a male patient aged 37 with unknown symptoms and travel history, swabbed from a lesion, region not reported, on 01 September 2022. To our knowledge, the five cases were not related to each other. Only the five genomes with rearrangements are described and discussed in this study.

## Results

**Fig. 1:**
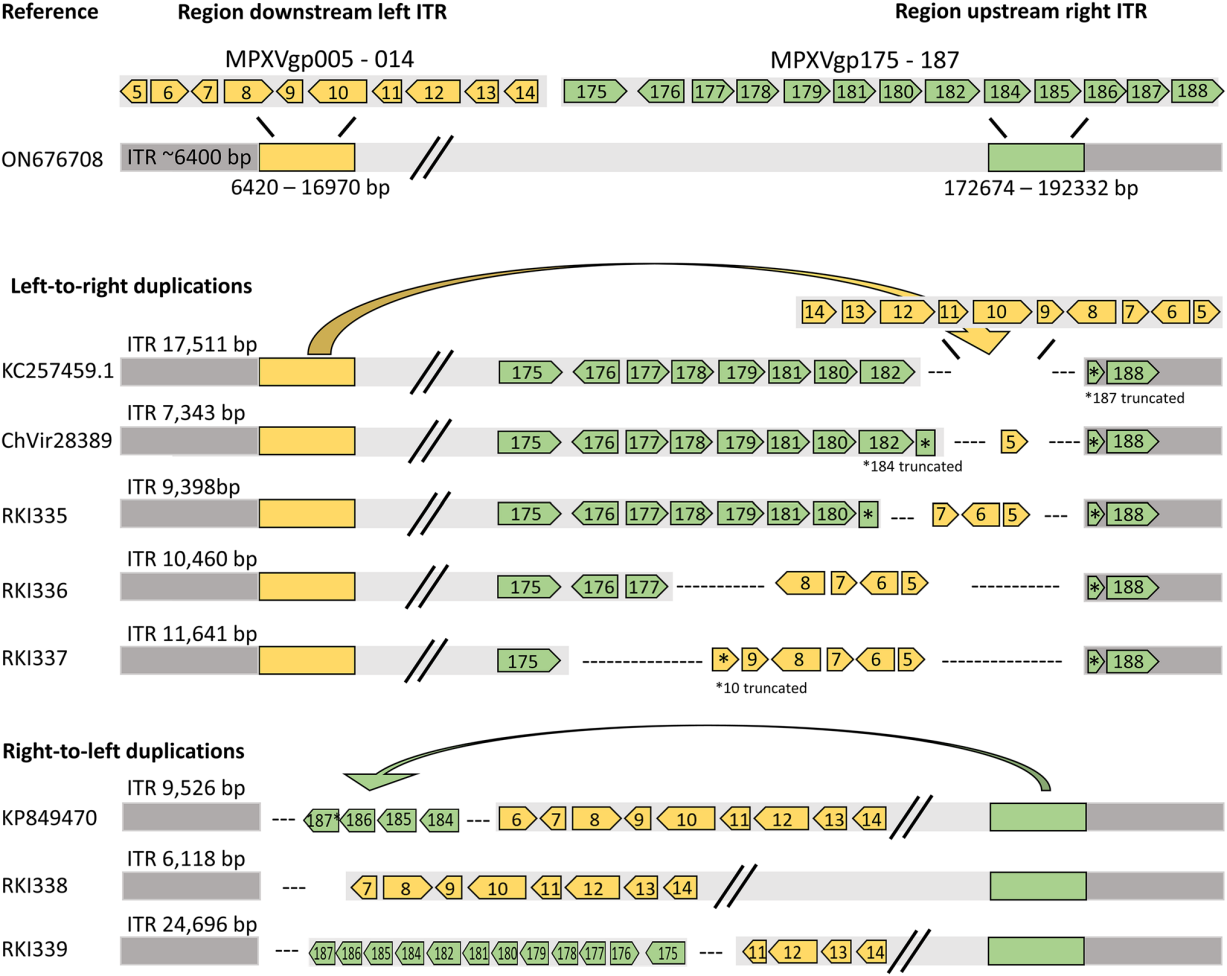
Genes affected by duplications and deletions for left-to-right (KC257459.1, MPXV/Germany/2022/RKI335, MPXV/Germany/2022/RKI336, MPXV/Germany/2022/RKI337 and ChVir28389) and right-to-left transitions (KP849470.1 as a representative for 20 genomes of MPXV clade II from 1958 to 2018, MPXV/Germany/2022/RKI338, MPXV/Germany/2022/RKI339). For left-to-right transitions, the region directly downstream the left ITR, always including MPXVgp005 and several of the genes MPXVgp006—MPXVgp014, is copied reverse complementary to upstream the right ITR, resulting in the extension of the ITR regions. The duplication to the region results in different sizes of deletion, always including a part of MPXVgp187 and the genes MPXVgp175—MPXVgp186. For right-to-left transitions, the partial MPXV187 and several of the genes MPXVgp175—MPXVgp186 are copied reverse complementary to the left side of the genome, resulting in increased length of the ITR regions and deletion of the genes MPXVgp005—MPXVgp010. One of the genomes only shows deletion of the regions MPXV005 and MPXV 006, without right-to-left duplication. Dark grey: ITR; Yellow: Region directly downstream the left ITR, duplicated to the region upstream the right ITR for left-to-right duplications; Green: Region directly upstream the right ITR, duplicated to region downstream the left ITR for right-to-left duplications; "---": Region deleted after introduction of duplication

Whole genome sequencing and de novo assembly of 339 genomes of the current multi-country outbreak of Mpox revealed five genomes with duplications, insertions and deletions of a similar pattern, which are reported in this study (Fig. 1, Table 2). These five genomes were assigned to clade IIb, lineage B.1, with up to three private mutations to reference ON676708.

**Table 2:** Duplication and deletion sites and length for left-to-right (KC257459.1, MPXV/Germany/2022/RKI335, MPXV/Germany/2022/RKI336, MPXV/Germany/2022/RKI337, ChVir28389) and right-to-left transitions (KP849470.1 as a representative for 20 genomes of MPXV clade II from 1958 to 2018, MPXV/Germany/2022/RKI338, MPXV/Germany/2022/RKI339)

	Duplication start (bp)	Duplication end (bp)	Deletion start (bp)	Deletion end (bp)	Duplication length (bp)	Deletion length (bp)	ITR length (bp)	Genome length (bp)
KC257459.1	6420	16,970	18,644	190,752	10,550	2108	17,511	206,372
MPXV/Germany/2022/RKI335	6420	9341	181,859	190,752	2,921	8893	9,398	191,176
MPXV/Germany/2022/RKI336	6420	10,460	175,449	190,752	4,040	15,303	10,460	185,852
MPXV/Germany/2022/RKI337	6420	11,702	173,826	190,752	5,282	16,926	11,641	185,251
ChVir28389	6420	7278	188,704	190,752	858	2048	7341	196,039
KP849470.1	188,453	190,752	6420	7076	2,299	4777	9526	200,397
MPXV/Germany/2022/RKI338			6420	8729		2309	6118	194,616
MPXV/Germany/2022/RKI339	172,538	190,752	6420	13,121	18,214	6701	24,695	208,804

Private mutations to reference ON676708.

MPXV/Germany/2022/RKI335 showed the mutations MPXVgp078:S211L and MPXVgp118:D653N to reference ON676708 (isolate MPXV_USA_2021_MD), which were both shared with two of the 339 sequenced genomes. Interestingly, proteins 78 and 118 are both transcription factors. MPXV/Germany/2022/RKI336 showed no private mutations to reference ON676708. MPXV/Germany/2022/RKI337 had the mutations MPXVgp029:E42K, MPXVgp167:P221S and MPXVgp010:P268Stop, shared with 19, two and one of the 339 sequenced genomes, respectively. Protein 29 is an IFN resistance PKR/eIF-alpha inhibitor, protein 167 a membrane glycoprotein and protein 10 an ankyrin/host range protein. MPXV/Germany/2022/RKI338 showed the mutation MPXVgp182:S750L, shared with none of the 339 sequenced genomes. Protein 182 is the B21R surface glycoprotein. MPXV/Germany/2022/RKI338 showed the mutations MPXVgp022:R111K and MPXVgp141:E119K, shared with none and one of the 339 sequenced genomes, respectively. Proteins 22 and 141 are a putative TLR signaling inhibitor and an RNA polymerase subunit.

Gene duplications and deletions.

Three of the five genomes revealed duplications from the left end to the right end with deletions at the site of insertion. Two of the five genomes showed deletions of genes at the left end, with one genome also having a duplication from the right to the left end.

Genome MPXV/Germany/2022/RKI335 showed a duplication from the region downstream the left ITR, including the genes MPXVgp005–007, to the regions upstream the right ITR between genes MPXVgp182 and MPXVgp188, resulting in truncation of MPXVgp184 and MPXVgp187 and complete deletion of MPXVgp185–186. The size of duplication (2921 bp) to the right end and the deletion of 8,893 bp resulted in a total genome length of 191,176 bp (ON676708: 197,173 bp) and the extension of the ITR regions from ~ 6400 bp to 9398 bp. Similar duplications and deletions were shown for MPXV/Germany/2022/RKI336 with a duplication of the genes MPXVgp005–008 and insertion at the right genome end between MPXVgp177 and MPXVgp188, with truncation of MPXVgp187 and complete deletion of genes MPXVgp178–186. The duplication of 4040 bp and deletion of 15,303 bp resulted in a genome length of 185,852 bp with ITRs of 10,460 bp. Genome 3 (MPXV/Germany/2022/RKI337) showed the same pattern of duplication and deletion, with the largest duplication of 5,282 bp (MPXVgp005—MPXVgp010 [truncated]) to the right end, with also the largest deletion of 16,926 bp (MPXVgp176—MPXVgp187 [truncated]). The genome length of MPXV/Germany/2022/RKI337 was 185,251 bp with ITRs of 11,641 bp.

Performing whole genome alignments with all of the MPXV genomes available at NCBI GenBank, such duplications from left-to-right were also found in a clade I MPXV genome from 2005 (KC257459.1, Monkeypox strain Sudan 2005) and in a genome from the recent outbreak (hMPXV/Germany/BE-ChVir28389/2022|EPI_ISL_13890482|2022-06-08) recently published [36, 37]. The MPXV genome from the Sudan showed a duplication of 10,550 bp from the same region after the left ITR to the right ITR, with a deletion of 2108 bp. The recently described duplication in genome E-ChVir28389 showed a duplication of 858 bp from the left to the right ITR, with a deletion of 2048 bp.

Duplications from the right to the left end were also revealed, with MPXV/Germany/2022/RKI339 having the largest duplication of 18,214 bp (genes MPXVgp174 [truncated]—MPXVgp187 [truncated]) to the left end, resulting in the deletion of genes MPXVgp005—MPXVgp010 (6,701 bp). The genome length was increased to 208,804 bp with extended ITRs of 24,695 bp. Genome MPXV/Germany/2022/RKI338 showed a deletion of 2309 bp in the same region downstream the left ITR, but without an insertion. Similar duplications were noticed in 20 genomes of clade II MPXV from 1958 to 2018.

For left-to-right duplications, the length of the duplications could be correlated with increased length of deletions at the insertion site and increased length of ITR regions.

## Discussion

During the SARS-CoV-2 pandemic, genomic surveillance has been focusing on single non-synonymous mutations, arising frequently and resulting in different phenotypes and variants and affecting virulence and host escape [38]. Poxviruses evolve much slower than RNA viruses like SARS-CoV-2, and it is rather hypothesized that all of today’s OPXVs descended from a common ancestor and evolved by loss of accessory genes, including those relevant for host adaption [39]. Deleted genes could be correlated with loss of an antigenic signal or continued evolutionary persistence by the attenuation of disease [40]. In contrast, acquired genes may support evasion of host defenses. For example, “genome accordions” have been described as a rapid mechanism for adaption of VACV, including amplification of genes under selective pressure, accumulation of point mutations and the following deletion of the additional genes [34]. CPXV has the highest number of accessory genes and the broadest host range of all known OPXV, while VARV has the smallest set of such genes and no natural host other than humans [41]. VARV strains from human archeological remains from the Viking Age were possibly widespread, but less pathogenic, supported by the discovery of accessory genes which are lost in modern-day VARV [35]. It is hypothesized that the variola-like ancestors’ host reservoir were rodents, as described for many modern OPXVs today, including MPXV, and evolved into modern highly pathogenic VARV after spill-over and adaption to the human host [42]. Gene gain and loss have been described previously for MPXV. In 60 samples from humans in DRC, collected between 2005 and 2007, 10 MPXV genomes showed a 625-bp deletion directly upstream the right ITR, as shown for the left-to-right transitions in our study, although no gene insertion could be observed. The deletion included one full and one truncated gene and seemed to correlate with human-to-human transmission [33]. Furthermore, as shown in Fig. 1, 20 clade II genomes obtained from GenBank contained duplications from right to left. Most of the genomes were derived from chimpanzees sampled in 2017 and rodents from the US outbreak in 2003 [43, 44]. The Sudan clade I genome with the largest insertion, KC257459.1, was sampled from a human Mpox outbreak in the Southern Sudan in 2005 [37]. Although sequences were obtained directly from primary clinical material, it is mentioned that the insertion was partially lost after the second passage in cell culture.

We could show similar left-to-right and right-to-left duplications, resulting in large deletions in five genomes of the 2022 human Mpox outbreak. All left-to-right duplications included one or more of the genes MPXVgp005—MPXV014, with deletion of one or more of the genes MPXVgp175—MPXVgp187, or the other way around for right-to-left duplications. For all left-to-right duplications, at least genes MPXVgp005—MPXVgp007 were included. Hypothesized protein functions are shown in supplementary table 1. MPXVgp005 is not homologous to any gene of the OPXV genus and its function is unknown. Furthermore, after reannotation of the genome, the fragmented gene was suggested to be removed [45]. MPXVgp006 is an epidermal growth factor-like protein, homologous to the C11R Vaccinia virus gene. It was shown that deletion of C11R reduced NF-κB activation in cell culture [46]. It has also been discussed whether MPXVgp007 has been annotated correctly [45], since it has no homologue in any other OPXV. Further duplicated genes are MPXVgp008 (zinc-finger protein, inhibition of apoptosis), MPXVgp009 (functional inhibitor of interleukin 18) and MPXVgp010 (truncated ankyrin/host range protein). In addition, all left-to-right duplications led to the deletion of at least the genes MPXVgp184 (truncated)—MPXVgp187 (truncated). Again, MPXVgp184 and MPXVgp186 have no OPXV homologues and are discussed to be annotated incorrectly [45]. MPXVgp185 is homologous to Vaccinia B22R, an immune-modulating gene with homology to serine protease inhibitors [47]. Deletion of B22R has been shown to decrease viral replication and virulence [48]. For MPXV/Germany/2022/RKI336 and MPXV/Germany/2022/RKI337 genes, MPXVgp180 (B19R) and MPXV182 (B21R) are further deleted—being two out of the six candidates identified as main possible virulence genes in MPXV [45]. Another one of these candidates, D10L (MPXVgp013), together with B19R and B21R, is duplicated in MPXV/Germany/2022/RKI339, and a fourth candidate is duplicated in the Sudan KC257459 genome shown in the results.

One may speculate whether such genome rearrangements lead to different MPXV phenotypes and are a sign of ongoing adaption to the human host. However, a small fraction of all sequenced genomes (1.5%) showed an unexpected flexibility of the genome structure, which needs to be monitored in future sequencing efforts. Relevant accessory host range genes are duplicated in the case of right-to-left duplications but are also deleted in the case of left-to-right duplications. Furthermore, the function of genes is often hypothesized or adopted from VACV or CPXV. Duplications near the termini have been described frequently for CPXV grown in chicken embryos, induced by crossover recombinational events between two genomes in opposite direction [49]. However, the large insertions are described to be unstable, being lost after passages in cell culture [50]. Similar intragenomic sequence transposition was shown for MPXV grown in chicken embryos, with large deletions and rearrangements in the ITR regions. The results suggested that terminal regions interact during MPXV replication to repair putative deletions [51]. The MPXV/Germany/2022/RKI338 only shows a deletion after possible loss of the insertion, and it has been described for VARV [35]. Another famous example is VACV which was used as a vaccine in the eradication of smallpox. VACV is thought to be derived from what today is known as horsepox virus, which has possibly been used by Edward Jenner when inventing vaccination [52, 53]. One of the main differences to today’s VACV are 10.7-kb and 5.5-kb insertions at the genomes’ ends. Vaccines from the US Civil War era (1859–1873) consist of true horsepox virus including the insertions, but also VACV-like viruses with and without the insertions as well as intermediates with only one insertion and end-to-end duplications, including a right-to-left duplication covering the MPXV homologue genes MPXVgp172—MPXVgp182 [54, 55]. Terminal genome deletions and insertions can also be identified between VACV and VARV [56]. Early VACV was disseminated through serial arm-to-arm transmission, with possible gene loss as an adaption to the host. The origin and natural host of the ancestor of VACV is still a mystery, but today’s VACV have only caused sporadic outbreaks and were not able to re-emerge in a natural host [57].

In four of the five genomes, the duplications led to expansions of the ITRs, with genome MPXV/Germany/2022/RKI339 having an ITR of 24,696 bp. ITR expansions have been described in myxoma viruses that were released in Australia [58]. The mechanism behind ITR expansions may involve nonhomologous recombination, probably as a mechanism to increase the gene dosage by gene duplication [59]. A model by Evans et al. describes the expansion of ITRs in different Vaccinia viruses, where ITR expansion has been observed, through nonhomologous recombination [60, 61]. Here, nonhomologous recombination between two double-stranded DNAs (virus A and virus B) in reverse orientation, with the junction at the region upstream the right ITR of virus A and a region downstream the left ITR of virus B, generates two recombined viruses: viruses A’ with missing genes downstream the junction at the right genome end, replaced by the inverted, extended left ITR of virus B. Virus B’ would have the deletion at the left ITR after the position of the junction, replaced by the extended right ITR of virus A. For our example MPXV/Germany/2022/RKI337, recombination may have occurred between viruses A and B in reverse orientation, with a junction between MPXVgp175 and of virus A and MPXVgp11 of virus B. This nonhomologous recombination would generate virus A’ with deletion of all genes after MPXVgp175, which are replaced by the inverted and extended left ITR of up to MPXVgp10 of virus B, which is MPXV/Germany/2022/RKI337. The hypothetical virus B’ would have all the genes of the right end of virus A (MPXVgp176-MPXVgp187) duplicated to its left end, with deletion of genes MPXVgp4-MPXVgp10, similar to MPXV/Germany/2022/RKI339.

In this study, we only found five out of 339 MPXV genomes (1.47%) with rearrangements. Ongoing sequencing efforts will have to show whether some of the described insertions and deletions will become fixed, or whether they are neutral or even disadvantageous for the virus. The MPXV of the 2022 outbreak may have become adapted to the human host, as continuous human-to-human transmission within a short time is seen for the first time since the discovery of MPXV in 1958. In addition, the mode of transmission and the phenotype of disease has changed [62, 63]. Although adaption to the host is not necessarily correlated with increased pathogenicity and virulence, it is not known during the ongoing Mpox outbreak how the virus will evolve in future. Intensified vaccination and changes in human behavior could exert selective pressure on the virus, with mutations, insertions and deletions being advantageous for transmission or immune escape. Although Mpox daily cases worldwide have decreased (as of January 16, 2023), it is likely that Mpox outbreaks will continue to appear, with different strains circulating [64].

## Conclusions

In the Mpox 2022 multi-country outbreak, rearrangements occur, including gene transitions to the opposite genome end and deletions at the insertion site of the genomes, and they should be monitored by whole genome sequencing in addition to single mutations.

## Supplementary Information

Below is the link to the electronic supplementary material.

Supplementary file1 (XLSX 12 KB)

## Data Availability

All genomes in this study were uploaded to NCBI GenBank (MPXV/Germany/2022/RKI335 – MPXV/Germany/2022/RKI339), accession numbers OP696838, OP696839, OP696840, OP696841 and OP696842. All sequence data are deposited under the project number PRJEB56807 at the European Nucleotide Archive.
